# Implementation Mapping to Identify Strategies to Increase Timely Postoperative Radiotherapy Initiation for Head/Neck Cancer

**DOI:** 10.1002/ohn.1268

**Published:** 2025-04-30

**Authors:** Laila A. Gharzai, Jaymie Bromfield, Michelle Kwan, Alexis Larson, Janine A. Kingsbury, Adil Akthar, Gaurava Agarwal, Julia H. Vermylen, Sara Becker, Kelli Scott, Amelia E. Van Pelt, Katelyn O. Stepan

**Affiliations:** ^1^ Department of Radiation Oncology Northwestern University Chicago Illinois USA; ^2^ Department of Medical Social Sciences Northwestern University Chicago Illinois USA; ^3^ Department of Otolaryngology Northwestern University Chicago Illinois USA; ^4^ Feinberg School of Medicine Northwestern University Chicago Illinois USA; ^5^ Department of Psychiatry Northwestern University Chicago Illinois USA; ^6^ Department of Medicine Northwestern University Chicago Illinois USA; ^7^ Department of Medical Education Northwestern University Chicago Illinois USA; ^8^ Center for Dissemination and Implementation Science Northwestern University Chicago Illinois USA

**Keywords:** clinical quality, head/neck cancer, PS/QI, radiotherapy

## Abstract

**Objective:**

Timely initiation of postoperative radiotherapy (PORT) for head and neck squamous cell carcinoma (HNSCC) is associated with improved survival, but rates of timely PORT initiation are low. To support uptake in a tertiary academic center, we aimed to identify implementation determinants (eg, barriers and facilitators) to timely PORT initiation and to design context‐specific implementation strategies.

**Methods:**

We created an implementation blueprint through a sequential mixed‐methods study where we (1) identified determinants by fielding a 15‐item survey based on the Theoretical Domains Framework (TDF), (2) prioritized determinants through focus groups with relevant stakeholders, (3) mapped barriers to implementation strategies using the Consolidated Framework for Implementation Research (CFIR)‐Expert Recommendations for Implementing Change (ERIC) matching tool, and (4) operationalized strategies using the Action, Actor, Context, Target, Time (AACTT) framework.

**Results:**

Twenty‐three participants from three departments (61% Radiation Oncology, 35% Otolaryngology, 4% Medical Oncology) in a variety of roles (35% physicians, 39% nurses or advanced practice providers, 22% radiation therapists or dosimetrists, and 4% research coordinators) completed surveys. Participants identified 10 determinants affecting timely PORT initiation. After strategy selection and operationalization by focus group participants (n = 13), three ERIC strategies were selected for clinical implementation: remind clinicians, conduct educational meetings, and facilitate relay of clinical data to providers.

**Discussion:**

This work developed a menu of implementation strategies for future deployment to support timely PORT initiation. Codesign centered the voice of frontline workers, increasing the likelihood of successful implementation.

**Implications for Practice:**

The systematic approaches to development can serve as a model for process improvement in other contexts.

Head and neck squamous cell carcinoma (HNSCC) is the sixth most common cancer in the world.^1^ This prevalence is expected to increase with the higher burden of human papillomavirus‐driven HNSCCs in developed countries.^2^ Evidence‐based interventions for HNSCC are multidisciplinary,^3^ with patients commonly undergoing a combination of surgery, radiotherapy, and chemotherapy. In patients with locally advanced HNSCC undergoing surgery, postoperative radiotherapy (PORT) is often recommended to reduce the risk of cancer recurrence. To maximize the oncologic benefits of PORT, timely initiation within 6 weeks of surgery is critical. The process of initiating PORT is complex and requires referrals to radiation oncology and dentistry, as well as multiple steps for generating radiotherapy plans before treatment starts.

Unfortunately, delays in the initiation of PORT are common across settings in the United States, which results in poorer health outcomes. National rates of timely initiation of PORT are approximately 40%.^4^ Studies have shown that patients who have timely completion of overall total treatment (time from surgery to completion of PORT) had double the rate of tumor control and overall survival as compared to those whose treatments were delayed,^5^ with an approximate 5% decrease in 5‐year overall survival per week that PORT is delayed.^6^ This suggests that PORT delay is associated with tumor recurrence, which directly impacts survival.^7^ Given these strong data, in 2021, the Commission on Cancer made timely PORT initiation the first quality metric in HNSCC care.^8^ Despite the known benefit, this large gap between real‐world initiation rates and quality recommendations^4^ suggests a strong need to better understand factors affecting the timely initiation of PORT.

Prior studies have identified a wide range of barriers to the timely initiation of PORT in HNSCC. Barriers include communication failures,^9^ inadequate education,^10^ dental challenges,^11^ and postoperative complications.^12^ The complex steps from surgery to PORT initiation can be exacerbated by chaotic work environments, administrative burdens, and time pressures, all of which are common drivers of burnout among clinicians,^13^, ^14^, ^15^ and thus PORT delays may contribute to provider dissatisfaction, moral distress, and burnout. Prior studies have employed both clinician‐ and patient‐directed strategies to improve timely initiation including integrating electronic order sets, improving referral patterns,^16^ and incorporating in‐person navigation to help patients schedule appropriate appointments.^17^ However, context is key, as implementation determinants and strategies depend on the setting in which PORT is implemented. Although data exist pointing to barriers and potential strategies to reduce PORT delays, more rigorous evaluations are needed across settings to identify optimal implementation strategies.

Implementation science is the use of intentional, proactive methods to integrate research into routine practice and can be harnessed to support the quality‐based goal of timely PORT initiation in HNSCC.^18^ Key methods that facilitate the integration of evidence‐based practices such as timely PORT initiation into routine clinical care are the use of conceptual frameworks to guide the implementation process,^19^, ^20^ identification of contextual factors that hinder or facilitate intervention uptake, selection of implementation strategies,^21^ and measurement of implementation outcomes.^22^, ^23^, ^24^ Guided by a systematic implementation science approach, this study employed mixed methods to first identify implementation determinants (ie, barriers and facilitators) associated with timely initiation of PORT for HNSCC and then designed context‐specific implementation strategies (ie, actions to increase uptake). Utilizing this input, this study aimed to develop an implementation blueprint outlining strategies for PORT initiation for patients with HNSCC in a tertiary academic center in a large, urban setting.

## Methods

We utilized a sequential mixed‐methods approach,^25^ first deploying a survey, which then guided qualitative focus groups seeking to build an implementation research logic model with specific strategies to improve rates of timely PORT initiation. In the first step, the Theoretical Domains Framework (TDF),^26^, ^27^ a framework synthesizing theories of behavior and behavior change related to the implementation of evidence‐based recommendations, informed the development of a survey seeking to identify barriers and facilitators to timely PORT initiation. In the second step, determinants were organized according to the dimensions of the Consolidated Framework for Implementation Research (CFIR),^20^ a determinant framework consisting of five domains across multiple ecological levels: outer setting, inner setting, innovation, individuals, and implementation process. Determinants were matched to implementation strategies listed in the Expert Recommendations for Implementing Change (ERIC)^21^ taxonomy. Each implementation strategy was then operationalized to be used clinically.^28^ This project was undertaken as a Quality Improvement project and as such does not constitute human subjects research; Northwestern University's Institutional Review Board (IRB) guidance on quality improvement and program evaluation projects is available upon request.

### Survey

**Figure 1:**
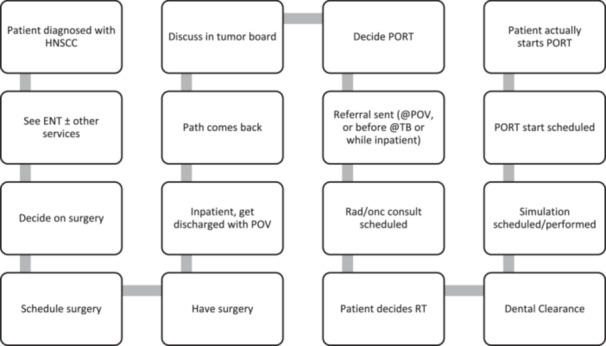
Process map. ENT, ear, nose, and throat; HNSCC, head and neck squamous cell carcinoma; path, pathology; PORT, postoperative radiotherapy; POV, post‐operative visit; RT, radiotherapy; TB, tumor board.

The anonymous 15‐item survey to assess barriers and facilitators to PORT initiation was developed by study team members (L.A.G., G.A., J.H.V., and K.O.S.) using a two‐step, theory‐guided method (see Supplemental Data S1, available online). First, a process map was developed by multidisciplinary study team members (L.A.G., J.A.K., A.L., and K.O.S.) describing the steps from patient diagnosis with HNSCC to delivery of the first PORT treatment (Figure 1). Steps identified in the process map were used to construct survey questions to facilitate a comprehensive assessment of 10 potential determinants (5 barriers and 5 facilitators), and these questions were then further refined using the TDF.^26^, ^27^ Items were generated from TDF behavior change constructs: knowledge (ie, knowledge about importance of timely PORT initiation and rates of completion); self‐efficacy (eg, areas of process under one's control); anticipated outcomes/attitude (eg, hectic/scrambling behaviors related to process); environmental constraints (eg, barriers/facilitators related to process); social influences (eg, communication between teams); emotion (eg, burnout, as measured by the emotional exhaustion question of the 2‐item Maslach Burnout Scale).^29^ To supplement these questions, two additional items on moral distress were adapted from the Moral Injury Symptom Scale^30^ assessing disappointment and loss of control, and two demographic questions (respondent's home department and clinical role).

All surveys were administered via an electronic link that was distributed in February 2024 at a weekly multidisciplinary head/neck tumor board meeting. The weekly tumor board is typically attended by approximately 20 to 30 members from the departments of Radiation Oncology, Otolaryngology, Medical Oncology, Radiology, and Pathology. Staff roles include physicians, nurses, advanced practice providers, trainees (residents and fellows), and research staff. Additional staff members who do not typically attend tumor boards (radiation therapists, radiation dosimetrists) but who do engage in routine care of patients with HNSCC were emailed separately inviting participation. The survey remained open for 5 days. Survey data were analyzed using descriptive statistics for categorical and continuous variables. Burnout was calculated as a binary variable, with those participants selecting once a week or more being considered as experiencing burnout.^31^

### Focus Groups

Focus groups were conducted to expand upon survey responses by prioritizing identified determinants, assessing additional determinants not reported on the surveys, identifying tentative implementation strategies to address timely PORT barriers, and operationalizing each strategy. The methodology and steps for the focus groups were developed by a multidisciplinary team that included two clinicians (L.A.G. and K.O.S.), two implementation scientists (K.S. and A.E.V.P.), and two additional clinicians with expertise in change management and physician wellness (G.A. and J.H.P.). The study group included clinicians directly embedded in the care of HNSCC, clinicians who do not treat cancer regularly, and social scientists without medical backgrounds, allowing for a diverse range of perspectives.

Focus group members were identified from a free‐text item in the survey asking for volunteers, and additional participants were purposefully selected to ensure diverse representation of the workforce (ie, to ensure all possible roles were represented). These additional participants may or may not have completed the prior anonymous survey. The Department of Otolaryngology has the following staff dedicated to the treatment of HNSCC: three physicians, two advanced practice providers, three nurses, and one research coordinator. The Department of Radiation Oncology has two physicians who specialize in the treatment of HNSCC, one nurse, and one advanced practice provider who provides additional support. In addition to this, general staff members who support the treatment of patients with HNSCC include a subset of three dosimetrists and approximately 10 radiation therapists who assist with simulations for radiotherapy planning and with the delivery of radiotherapy. Thus, the overall sampling method ensured a diverse representation of the overall workforce.

Each focus group was held in the Department of Radiation Oncology and Otolaryngology and lasted for approximately 1 hour. Because participants needed to prioritize their clinical duties and could not be available at the same time, multiple small groups were offered within each department. Focus groups were facilitated by the first and last author (L.A.G. and K.O.S.), a radiation oncologist and otolaryngologist, both of whom specialize in the treatment of HNSCC. For each step performed in the focus groups described below (logic model, determinant ranking, and operationalization), participants were provided with individual handouts for each step and were requested to complete each step by writing on the handouts. The logic model and strategy operationalization were completed together with one completed form for each focus group, and the determinant ranking was performed by each individual participant. Facilitators performed critical memoing during the discussion to ensure that critical themes and decisions were recorded. After completion of all the focus groups, the completed forms were later analyzed by the first and last author (L.A.G. and K.O.S.) with a rapid analytic approach to create a consensus document for the logic model and strategy operationalization.^32^

Determinants obtained from the survey were discussed, and participants collaboratively completed an implementation research logic model. An implementation research logic model is a tool that aims to assist researchers in planning, executing, reporting, and synthesizing implementation projects.^33^ Given the complexity of the PORT initiation process, a logic model helped structure focus group discussions and allowed for mapping of strategies to individual determinants. Determinants were placed within the five CFIR^20^ domains by the focus group participants. We elicited feedback to identify additional determinants that were not identified from the process map and survey. After placing determinants into CFIR domains, individual participants were asked to rank all determinants on a paper document where 1 indicated the most important. A summary score of the average rank of each determinant was created for the overall group.

For each determinant identified during the survey, context experts (L.A.G. and K.O.S.) developed a preliminary specific strategy (called “action step” hereafter) before the focus groups. Each preliminary action step was presented to focus group participants. Feedback was elicited, and the action step was refined. Each action step was operationalized by focus group members following Presseau et al's Action, Actor, Context, Target, Time (AACTT) framework.^28^ The AACTT framework aims to clearly provide descriptions and specifications of behaviors in implementation research.^28^ Following the focus groups, the action steps were reviewed with implementation science experts (K.S. and A.E.V.P.) to ensure appropriate specificity. The experts then used the CFIR‐ERIC^21^ matching tool, a widely used resource to support matching barriers to effective implementation strategies, to map determinants and specific action steps to broader ERIC strategies and finalize the implementation research logic model.

### Implementation Strategy Selection

After completion of the focus groups, team members met to review and identify strategies suitable for immediate deployment (L.A.G., A.L., J.A.K., and K.O.S.). Team members reviewed the average ranking of each determinant. The ERIC implementation strategies associated with these top three determinants through the CFIR‐ERIC matching tool were selected for immediate deployment. All additional determinants were reviewed to identify additional strategies that the team felt could feasibly be addressed with low burden to providers. Upon team review, two additional determinants for which strategies could be implemented with minimal workflow changes were selected, for a total of five determinants.

## Results

### Participant Characteristics

A total of 23 survey participants from three departments (61% Radiation Oncology, 35% Otolaryngology, and 4% Medical Oncology) in a variety of roles (35% physicians, 39% nurses or advanced practice providers, 22% radiation therapists or dosimetrists, and 4% research coordinators) contributed to the anonymous survey. A total of 13 team members participated in focus groups, 6 from Otolaryngology and 7 from Radiation Oncology, in a variety of roles (15% physicians, 46% nurses or advanced practice providers, 31% radiation therapists or dosimetrists, and 8% research coordinators). A total of four focus groups were held with focus group sizes ranging from 2 to 5. Focus group participants had a mean age of 35 years (standard deviation [SD] 12.2) and a median of 3 years (range 1‐20) experience working in their home department (Table 1).

**Table 1:** Demographics

	N (%)
*Survey*	23
Department	
Otolaryngology	8 (35%)
Radiation Oncology	14 (61%)
Medical Oncology	1 (4%)
Role	
Physician	8 (35%)
Nurse or advanced practice provider	9 (39%)
Radiation therapist or dosimetrist	5 (22%)
Research coordinator	1 (4%)
*Focus groups*	13
Department	
Otolaryngology	6 (46%)
Radiation Oncology	7 (54%)
Age (mean years, standard deviation)	35 (12.2)
Number of years worked in department (median, range)	3 (1‐20)
Role	
Physician	2 (15%)
Nurse or advanced practice provider	6 (46%)
Radiation therapist or dosimetrist	4 (31%)
Research coordinator	1 (8%)

### Survey: Identification of Implementation Determinants

Knowledge of the need for timely PORT initiation was high, with 91% strongly agreeing that starting PORT within 6 weeks was important utilizing a 5‐point Likert scale (1 = strongly disagree, 5 = strongly agree). Notably, 74% of participants overestimated institutional rates of timely initiation. The process of PORT initiation was perceived as hectic, with 91% of participants stating that they were scrambling in hectic or unplanned ways sometimes, usually, or always on a 5‐point scale where 1 = “never/almost never” and 5 = “always/almost always.” The complexity of the overall PORT process was rated at an average of 3.4 (SD 0.92), where 1 was very organized and 5 was hectic or requiring significant effort to start patients on time. When offered options for how the complexity of the PORT initiation process affected their workflows, the following options were selected: stressed/rushed workflows, overbooking patients, performing after‐hours work, and excessive messages (both between staff and to patients).

Communication and efficiency within and between teams were measured using a 5‐point Likert scale (poor, marginal, satisfactory, good, and optimal). Almost all participants felt that intradepartmental communication went well, with 91% stating that their team worked efficiently and effectively together (good or optimal). However, only 39% of participants stated that interdepartmental communication (between Radiation Oncology and Otolaryngology) demonstrated working well together (good or optimal). Nearly half of participants (48%) agreed or strongly agreed that they felt disappointed when they were unable to start PORT in a timely fashion, and 70% of participants agreed or strongly agreed that they felt a loss of control when scheduling challenges contributing to delayed PORT initiation occurred (using a 5‐point scale ranging from “strongly disagree” to “strongly agree”).

Overall burnout rate for the group, as described by those that selected once a week or more to the Maslach burnout question, was 35%. Those that were nonphysicians had a burnout rate of 53% as compared to 0% for physicians. Participants who experienced moral distress, as measured by disappointment and loss of control, had higher rates of burnout. Those who agreed or strongly agreed with feeling disappointed when PORT was delayed were over three times as likely to be burned out as compared to those who did not feel disappointed (55% vs 17%). Those who agreed or strongly agreed with feeling the loss of control when scheduling challenges occurred similarly had higher burnout (50%) as compared to those who did not feel loss of control (0%).

Building from the process map (Figure 1), participants selected barriers and facilitators to timely PORT initiation. The five barriers in priority order and the proportion of survey respondents endorsing each were the following: (1) dental clearance (100% of respondents), (2) postoperative complications (83% of respondents), (3) timing of referral to radiation oncology (74% of respondents), (4) communication between teams (52% of respondents), and (5) dosimetry (radiation planning) staff time (30% of respondents). The five facilitators in priority order were the following: (1) access to new radiation oncology consults (70% of respondents), (2) time to new radiation oncology simulation appointment (65% of respondents), (3) timely receipt of final pathology results (43% of respondents), (4) timely discussion of patients at tumor board (43% of respondents), and (5) time on radiation machine for new start (43% of respondents).

### Focus Groups: Elicitation of Implementation Strategies

**Figure 2:**
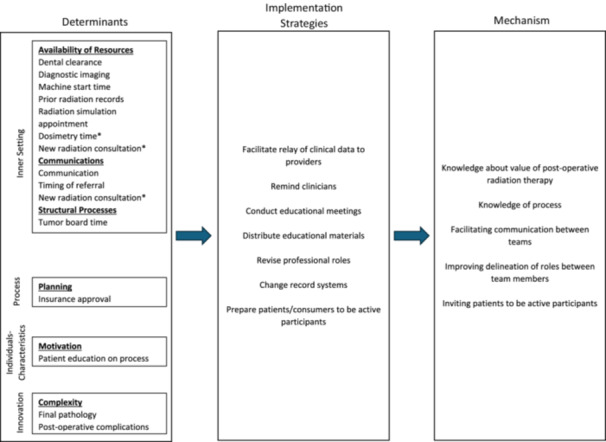
Simplified implementation research logic model. Asterisk demonstrates that two determinants were noted to represent two general constructs.

Focus group participants ranked all determinants, assigned the determinants into appropriate CFIR dimensions, and elicited additional determinants not identified through the survey (Table 2). Eight of the determinants were identified as inner setting and two as innovation. Four additional barriers were elicited during focus group discussions: two in the inner setting domain, one in the implementation process domain, and one in the individuals domain. These results were used to complete the implementation research logic model (Figure 2).

**Table 2:** Implementation Blueprint^a^

Rank	Determinant	Source	Responsible party	Implementation category^b^	Action step
1	Timing of referral	Survey	MD, APP	Remind clinicians	Send referrals before discharge after surgery.
2	Dental clearance	Survey	MD, APP, RN	Conduct educational meetings Remind clinicians	Establish cross‐departmental clearance guidelines.
3	Communication	Survey	MD, APP, RN, RTT, CMD	Facilitate relay of clinical data to providers	Improve intradepartmental communication.
4	Postoperative complications	Survey	MD, APP, RN	Facilitate relay of clinical data to providers Distribute educational materials Conduct educational meetings	Monitor symptoms and improve communication.
5	Final surgical pathology results	Survey	MD, APP, RN	Facilitate relay of clinical data to providers Conduct educational meetings	Educate team members and utilize the electronic medical record to flag results.
6	Dosimetry time	Survey	MD, RTT, CMD	Facilitate relay of clinical data to providers Revise professional roles	Efficiently utilize dosimetry time based on start date.
7	Radiation simulation appointments	Survey	MD, RTT, CMD	Remind clinicians	Continue scheduling simulations as soon as possible.
8	New radiation oncology consults	Survey	MD, APP, RN	Facilitate relay of clinical data to providers Change record systems	Continue seeing patients in a timely fashion and work toward multidisciplinary care with appointments with multiple specialties.
9	Tumor board review	Survey	MD, APP, RN	Facilitate relay of clinical data to providers	Flag patients when tumor board presentation delayed.
10	Start time on radiation machine	Survey	MD, RTT, CMD	Facilitate relay of clinical data to providers	Continue scheduling treatment start times at simulation.
Not ranked	Availability of diagnostic imaging	Focus groups	MD, APP, RN	Facilitate relay of clinical data to providers	Discuss patients at time of referral for additional workup needed.
Not ranked	Prior radiation records	Focus groups	MD, APP, RN	Facilitate relay of clinical data to providers	Request prior records when patient is scheduled for consultation.
Not ranked	Insurance approval	Focus groups	MD, APP, RN	Remind clinicians	Fill out insurance forms in a timely fashion.
Not ranked	Patient education on process	Focus groups	MD, APP, RN	Distribute educational materials Prepare patients/consumers to be active participants	Provide patients with additional education and pamphlets.

^a^ Team members: APP, advanced practice provider; CMD, certified medical dosimetrist; MD, medical doctor; RN, registered nurse; RTT, radiation therapy therapist.

^b^ Implementation categories constructed from the Consolidated Framework for Implementation Research (CFIR)‐Expert Recommendations for Implementing Change (ERIC) matching tool.

### Implementation Strategies

The CFIR‐ERIC matching tool highlighted a total of seven strategies to be implemented to address the 10 determinants and improve timely PORT initiation (Table 2). The most recommended strategy was to “facilitate relay of clinical data to providers,” which was recommended for nine determinants. The next most recommended strategies were “remind clinicians” (four determinants), “conduct educational meetings” (three determinants), and “distribute educational materials” (two determinants). The following strategies were recommended for one determinant each: “revise professional roles,” “change record systems,” and “prepare patients/consumers to be active participants.” In the section below, we describe the strategies generated to address the top three ranked determinants from the survey (radiation oncology referral, dental clearance, and communication within teams). In addition, we include strategies designed to address two determinants that were perceived as highly feasible to implement by the research team, one of which was in the survey (timely tumor board review, initially ranked as the ninth priority) and one of which was elicited during the focus group discussions (availability of prior radiation records). For each ERIC strategy, we discuss how it was operationalized and deployed in the clinic setting in this tertiary academic center.

To contend with the timing of radiation oncology referral (from Otolaryngology), the ERIC strategy was to “remind clinicians.” The specific action step generated was to “send referrals before hospital discharge after surgery.” After discussion with relevant providers, the workflow was modified to include a referral to radiation oncology sent before the patient's discharge from the hospital. Provider education was performed to ensure that referrals were sent both as a dedicated order and separately as an in‐basket message to the radiation oncology nursing team to schedule the patient for a visit.

To address dental clearance, the two ERIC strategies were “remind clinicians” and “conduct educational meetings.” The specific action step selected was to establish cross‐departmental guidelines for dental clearance, as well as guidelines for inpatient dental clearance. To facilitate this, study team members (L.A.G. and K.O.S.) met with the institution's lead dentist to discuss the feasibility of increasing the number of inpatient dental referrals, which was received positively. Additionally, an order set within the electronic medical record specific to complex HNSCC surgeries (free flaps) was modified to include dental clearance as an order to facilitate inpatient referrals.

To tackle communication between teams, the ERIC strategy was to “facilitate the relay of clinical data to providers.” The specific action step was to improve interdepartmental communication. Recommendations were to encourage closed‐loop communication, to include radiation oncology team members in a routine monthly otolaryngology check‐in meeting, and to host regular team‐building activities. The first social event hosted between the two teams (Radiation Oncology and Otolaryngology) enabled team members who routinely spoke on the phone or via messaging systems to meet in person for the first time. During this meeting, team members described standard workflows to enhance understanding across departments. Discussion included typical daily schedules for team members, providing guidance on when to expect responses to messages, when team members are typically on computers, other roles and responsibilities delegated to team members separate from HNSCC care (eg, rooming patients or providing outpatient anesthesia support), and other noncancer patient types that are typically managed by the teams. This discussion, along with the social aspect of the meeting, enhanced team members’ understanding of the roles and responsibilities of members of other departments and improved expectations for communication across the teams.

To address timely tumor board review, the ERIC strategy was to “facilitate the relay of clinical data to providers.” The standard clinical workflow for placing a patient on the tumor board for a given week was written communication from a provider to a research coordinator. This workflow was modified such that the research coordinator now monitors when the patient's surgery date is in relation to the upcoming scheduled tumor board presentation. For cases where the patient's tumor board presentation will be 3 weeks or more after surgery, the research coordinator in charge of sending out the tumor board list will notify the patient's treating otolaryngologist. This reminder will allow the treating physician to send a referral to radiation oncology sooner (rather than waiting until after tumor board) but is limited by requiring a person to remember to notify a physician.

Finally, to address the lack of availability of prior radiation records, the ERIC strategy was to “facilitate the relay of clinical data to providers.” The specific action step generated was to request prior radiation records once the patient is scheduled for a consultation (instead of waiting until after the patient is seen). For the team treating HNSCC within the Radiation Oncology department, the standard scheduling workflow includes asking patients if they have undergone prior radiation. Workflow has now been modified such that if patients respond affirmatively (that they have undergone prior radiation), records are now requested before the consultation appointment with the physician. This decreases delays related to waiting for the records after the consultation to render decisions on suitability for radiation.

## Discussion

We described the development of an implementation blueprint aiming to improve rates of timely PORT initiation for HNSCC in an academic setting. We created an implementation research logic model, ranked determinants, identified multiple potential strategies, and selected five specific strategies for active implementation into practice. We demonstrated how implementation science methods can be applied to solve clinical challenges, providing valuable insights into implementation determinants and strategies to improve uptake of timely PORT initiation.

Timely initiation of PORT for HNSCC is a prime example of an evidence‐based practice that is challenging to integrate into routine clinical care with multiple downstream adverse impacts on patient care and provider well‐being when not optimized.^4^, ^5^, ^6^, ^8^ Prior work has investigated many of the common themes that we also identified, such as prior work that has sought to improve rates of timely referrals to radiation oncology^11^ or improve coordination of dental care.^34^ Additional quality improvement efforts have sought to integrate dedicated staff members as patient navigators to address barriers.^16^, ^17^, ^35^ However, the clinical workflow at our tertiary medical center does not include a dedicated patient navigator or staff member available to manage an individual patient's challenges. Through this framework‐guided mixed‐methods approach, we demonstrated how implementation science methods can be used to identify strategies that are feasible to implement within the context of a specific practice.

Our survey guided by the TDF demonstrated a clear connection between provider burnout and clinical workflow. Survey participants who experienced more moral distress related to this hectic process of PORT initiation were more likely to report burnout as compared to those that did not experience moral distress. Thus, addressing clinical workflow challenges may offer a mechanism to address broader provider well‐being in addition to patient care quality improvement. Implementation science methods may allow for solutions bridging provider well‐being, clinical workflow challenges, and patient care gaps.

Our strategy selection process focused on the top three ranked determinants, as well as two additional determinants that could be most easily addressed by study team members. This process ultimately identified five strategies seeking to improve timely PORT initiation while balancing the capacity of staff to enact the changes required to implement the strategies. Dental care, one of the determinants selected, was a particularly thorny challenge to address. Patients are recommended to undergo dental extractions to minimize the risk of osteoradionecrosis posttreatment.^36^ However, this can be logistically difficult for patients to complete on time and is a significant financial burden.^37^ Although our strategy focused on areas within our circle of control, specifically on increasing inpatient dental clearance, additional work is needed to address this barrier in other contexts such as outpatient settings in a manner that minimizes the burden on patients.

HNSCC is associated with significant disparities; patients with lower socioeconomic status, those of non‐white ethnicity, and those with differing insurance coverage have been shown to be less likely to travel to a tertiary care center for treatment,^38^ more likely to undergo unplanned treatment breaks,^39^ and less adherent to follow‐up guidelines.^40^ HNSCC patients with higher financial burden have also been shown to require more supportive measures^41^ and potentially experience worse survival.^42^, ^43^ Thus, ensuring that the strategies generated here enhance health equity and do not exacerbate underlying disparities for minoritized groups is of critical importance moving forward. Future efforts to test the impact of identified implementation strategies on implementation outcomes (eg, reach, adoption, and feasibility) in this setting will need to ensure equity given the potential for exacerbating health disparities.

Our study focused only on the clinical workflows and processes leading to PORT delay, and thus included clinical providers engaged in the treatment of patients with HNSCC. Patients also experience multiple challenges that can limit timely PORT initiation. Understanding the barriers that patients themselves face, such as challenges related to transportation,^44^ will generate additional patient‐facing determinants that can be addressed for a broader effort seeking to develop additional multifaceted implementation strategies that improve patient care. Future work will need to integrate patient voices to identify determinants that prevent timely PORT initiation, and administrative voices who generate policies that impact PORT coordination.

This research has multiple limitations. First, the strategies identified here are limited to the context in which they were identified and may not generalize beyond an urban academic hospital. However, the implementation framework and methods used here can be expanded into other settings for additional contexts. Second, the strategies were specifically for patients who underwent surgery and subsequent PORT at the same institution. However, many of our patients are unable to come to our center for multiple weeks of PORT, and thus many receive PORT elsewhere. A multitude of challenges arise when patients undergo PORT either at an affiliate site or outside facility, and additional work is needed to capture mechanisms to address these specific challenges. Future efforts to reach a broader group of patients will include expanding this effort to network sites to incorporate patients who receive care at nonacademic medical centers. Third, the two departments in this setting were smaller, and a limited number of participants were included in the survey and focus groups; this may not be applicable to larger centers with more extensive departments. Finally, we only generated strategies to address five determinants due to the bandwidth of the clinical team. Future work might seek to generate strategies to address the other determinants identified in the survey and focus group.

In conclusion, we created an implementation blueprint to understand the determinants of timely PORT initiation in HNSCC. We identified five strategies to implement clinically, and efforts to measure implementation outcomes are ongoing. Future efforts seeking to continue to optimize PORT for patients with HNSCC are critical, given the known survival advantages for timely PORT initiation.

## Author Contributions

**Laila A. Gharzai**, conception, design, data acquisition/analysis/interpretation, drafting the work, final approval, and agreement to be accountable; **Jaymie Bromfield**, data analysis/interpretation, critically editing the work, final approval, and agreement to be accountable; **Michelle Kwan**, data analysis/interpretation, critically editing the work, final approval, and agreement to be accountable; **Alexis Larson**, data acquisition/analysis/interpretation, critically editing the work, final approval, and agreement to be accountable; **Janine A. Kingsbury**, data acquisition/analysis/interpretation, critically editing the work, final approval, and agreement to be accountable; **Adil Akthar**, data analysis/interpretation, critically editing the work, final approval, and agreement to be accountable; **Gaurava Agarwal**, data analysis/interpretation, critically editing the work, final approval, and agreement to be accountable; **Julia H. Vermylen**, data analysis/interpretation, critically editing the work, final approval, and agreement to be accountable; **Sara Becker**, data analysis/interpretation, critically editing the work, final approval, and agreement to be accountable; **Kelli Scott**, data analysis/interpretation, critically editing the work, final approval, and agreement to be accountable; **Amelia E. Van Pelt**, data analysis/interpretation, critically editing the work, final approval, and agreement to be accountable; **Katelyn O. Stepan**, conception, design, data acquisition/analysis/interpretation, drafting the work, final approval, and agreement to be accountable.

## Disclosures

### Competing interests

None.

### Funding source

Laila A. Gharzai and Katelyn O. Stepan report funding from Northwestern University's Scholars of Wellness program supporting the current work. Grant number N/A.

## Supporting information

Supplement: Anonymous 15‐item survey to assess barriers and facilitators to PORT initiation developed by study team members and fielded to members in the Departments of Radiation Oncology, Otolaryngology, and Medical Oncology.

## References

[1] Siegel RL , Giaquinto AN , Jemal A . Cancer statistics, 2024. CA Cancer J Clin. 2024;74(1):12‐49. 10.3322/caac.21820 38230766

[2] Tota JE , Best AF , Zumsteg ZS , Gillison ML , Rosenberg PS , Chaturvedi AK . Evolution of the oropharynx cancer epidemic in the united states: moderation of increasing incidence in younger individuals and shift in the burden to older individuals. J Clin Oncol. 2019;37(18):1538‐1546. 10.1200/JCO.19.00370 31026209 PMC6599405

[3] Caudell JJ , Gillison ML , Maghami E , et al. NCCN Guidelines® insights: head and neck cancers, version 1.2022: featured updates to the NCCN Guidelines. J Natl Compr Cancer Netw. 2022;20(3):224‐234. 10.6004/jnccn.2022.0016 35276673

[4] Lorenz FJ , Mahase SS , Miccio J , King TS , Pradhan S , Goyal N . Update on adherence to guidelines for time to initiation of postoperative radiation for head and neck squamous cell carcinoma. Head Neck. 2023;45(7):1676‐1691. 10.1002/hed.27380 37102787 PMC10797635

[5] Ang KK , Trotti A , Brown BW , et al. Randomized trial addressing risk features and time factors of surgery plus radiotherapy in advanced head‐and‐neck cancer. Int J Radiat Oncol Biol Phys. 2001;51(3):571‐578. 10.1016/s0360-3016(01)01690-x 11597795

[6] Harris JP , Chen MM , Orosco RK , Sirjani D , Divi V , Hara W . Association of survival with shorter time to radiation therapy after surgery for US patients with head and neck cancer. JAMA Otolaryngol Head Neck Surg. 2018;144(4):349‐359. 10.1001/jamaoto.2017.3406 29522072 PMC5876822

[7] Peters LJ , Goepfert H , Ang KK , et al. Evaluation of the dose for postoperative radiation therapy of head and neck cancer: first report of a prospective randomized trial. Int J Radiat Oncol Biol Phys. 1993;26(1):3‐11. 10.1016/0360-3016(93)90167-t 8482629

[8] Graboyes EM , Divi V , Moore BA . Head and neck oncology is on the national quality sidelines no longer‐put me in, coach. JAMA Otolaryngol Head Neck Surg. 2022;148(8):715‐716. 10.1001/jamaoto.2022.1389 35708673 PMC9378525

[9] Sykes KJ , Morrow E , Smith JB , et al. What is the hold up?‐Mixed‐methods analysis of postoperative radiotherapy delay in head and neck cancer. Head Neck. 2020;42(10):2948‐2957. 10.1002/hed.26355 33174308

[10] Graboyes EM , Halbert CH , Li H , et al. Barriers to the delivery of timely, guideline‐adherent adjuvant therapy among patients with head and neck cancer. JCO Oncol Pract. 2020;16(12):e1417‐e1432. 10.1200/OP.20.00271 32853120 PMC7735037

[11] Divi V , Chen MM , Hara W , et al. Reducing the time from surgery to adjuvant radiation therapy: an institutional quality improvement project. Otolaryngol Head Neck Surg. 2018;159(1):158‐165. 10.1177/0194599818768254 29631478

[12] Noyes EA , Burks CA , Larson AR , Deschler DG . An equity‐based narrative review of barriers to timely postoperative radiation therapy for patients with head and neck squamous cell carcinoma. Laryngoscope Investig Otolaryngol. 2021;6(6):1358‐1366. 10.1002/lio2.692 PMC8665479 34938875

[13] Shanafelt TD , Mungo M , Schmitgen J , et al. Longitudinal study evaluating the association between physician burnout and changes in professional work effort. Mayo Clin Proc. 2016;91(4):422‐431. 10.1016/j.mayocp.2016.02.001 27046522

[14] Sinsky CA , Willard‐Grace R , Schutzbank AM , Sinsky TA , Margolius D , Bodenheimer T . In search of joy in practice: a report of 23 high‐functioning primary care practices. Ann Fam Med. 2013;11(3):272‐278. 10.1370/afm.1531 23690328 PMC3659145

[15] Beltràn Ponce S , Small CJ , Amini A , et al. Overcoming burnout and promoting wellness in radiation oncology: a report from the ACR Commission on Radiation Oncology. J Am Coll Radiol. 2023;20(5):487‐493. 10.1016/j.jacr.2023.03.003 36925094 PMC10149602

[16] Voora RS , Stramiello JA , Sumner WA , et al. Quality improvement intervention to reduce time to postoperative radiation in head and neck free flap patients. Head Neck. 2021;43(11):3530‐3539. 10.1002/hed.26852 34492135

[17] Graboyes EM , Sterba KR , Li H , et al. Development and evaluation of a navigation‐based, multilevel intervention to improve the delivery of timely, guideline‐adherent adjuvant therapy for patients with head and neck cancer. JCO Oncol Pract. 2021;17(10):e1512‐e1523. 10.1200/OP.20.00943 33689399 PMC8791819

[18] Bauer MS , Damschroder L , Hagedorn H , Smith J , Kilbourne AM . An introduction to implementation science for the non‐specialist. BMC Psychol. 2015;3(1):32. 10.1186/s40359-015-0089-9 26376626 PMC4573926

[19] Flottorp SA , Oxman AD , Krause J , et al. A checklist for identifying determinants of practice: a systematic review and synthesis of frameworks and taxonomies of factors that prevent or enable improvements in healthcare professional practice. Implement Sc. 2013;8:35. 10.1186/1748-5908-8-35 23522377 PMC3617095

[20] Damschroder LJ , Reardon CM , Widerquist MAO , Lowery J . The updated Consolidated Framework for Implementation Research based on user feedback. Implement Sc. 2022;17(1):75. 10.1186/s13012-022-01245-0 36309746 PMC9617234

[21] Waltz TJ , Powell BJ , Fernández ME , Abadie B , Damschroder LJ . Choosing implementation strategies to address contextual barriers: diversity in recommendations and future directions. Implement Sci. 2019;14(1):42. 10.1186/s13012-019-0892-4 31036028 PMC6489173

[22] Proctor E , Silmere H , Raghavan R , et al. Outcomes for implementation research: conceptual distinctions, measurement challenges, and research agenda. Adm Policy Ment Health Ment Health Ser Res. 2011;38(2):65‐76. 10.1007/s10488-010-0319-7 PMC3068522 20957426

[23] Lewis CC , Mettert KD , Dorsey CN , et al. An updated protocol for a systematic review of implementation‐related measures. Syst Rev. 2018;7(1):66. 10.1186/s13643-018-0728-3 29695295 PMC5918558

[24] Mettert K , Lewis C , Dorsey C , Halko H , Weiner B . Measuring implementation outcomes: an updated systematic review of measures' psychometric properties. Implement Res Pract. 2020;1:2633489520936644. 10.1177/2633489520936644 37089128 PMC9924262

[25] Palinkas LA , Horwitz SM , Chamberlain P , Hurlburt MS , Landsverk J . Mixed‐methods designs in mental health services research: a review. Psychiatr Serv. 2011;62(3):255‐263. 10.1176/ps.62.3.pss6203_0255 21363896

[26] Michie S . Making psychological theory useful for implementing evidence based practice: a consensus approach. Qual Saf Health Care. 2005;14(1):26‐33. 10.1136/qshc.2004.011155 15692000 PMC1743963

[27] Atkins L , Francis J , Islam R , et al. A guide to using the Theoretical Domains Framework of behaviour change to investigate implementation problems. Implement Sci. 2017;12(1):77. 10.1186/s13012-017-0605-9 28637486 PMC5480145

[28] Presseau J , McCleary N , Lorencatto F , Patey AM , Grimshaw JM , Francis JJ . Action, Actor, Context, Target, Time (AACTT): a framework for specifying behaviour. Implement Sci. 2019;14(1):102. 10.1186/s13012-019-0951-x 31806037 PMC6896730

[29] Li‐Sauerwine S , Rebillot K , Melamed M , Addo N , Lin M . WestJEM 21.3 May Issue. West J Emerg Med. 2020;21(3):610‐617. 10.5811/westjem.2020.2.45139 32421508 PMC7234685

[30] Mantri S , Lawson JM , Wang Z , Koenig HG . Identifying moral injury in healthcare professionals: the moral injury symptom scale‐HP. J Relig Health. 2020;59(5):2323‐2340. 10.1007/s10943-020-01065-w 32681398 PMC7366883

[31] Dolan ED , Mohr D , Lempa M , et al. Using a single item to measure burnout in primary care staff: a psychometric evaluation. J Gen Intern Med. 2015;30(5):582‐587. 10.1007/s11606-014-3112-6 25451989 PMC4395610

[32] Nevedal AL , Reardon CM , Opra Widerquist MA , et al. Rapid versus traditional qualitative analysis using the Consolidated Framework for Implementation Research (CFIR). Implement Sci. 2021;16(1):67. 10.1186/s13012-021-01111-5 34215286 PMC8252308

[33] Smith JD , Li DH , Rafferty MR . The implementation research logic model: a method for planning, executing, reporting, and synthesizing implementation projects. Implement Sci. 2020;15(1):84. 10.1186/s13012-020-01041-8 32988389 PMC7523057

[34] Rosas Herrera AM , Haskins AD , Hanania AN , et al. Timely delivery of PORT for head and neck squamous cell carcinoma in a county hospital. Laryngoscope Investig Otolaryngol. 2024;9(1):e1211. 10.1002/lio2.1211 PMC10866599 38362185

[35] Graboyes EM , DeMass R , Sterba KR , et al. Randomized trial of enhanced versus standard navigation to promote timely initiation of adjuvant radiotherapy for head and neck cancer. JCO Oncol Pract. 2025:OP2400901. 10.1200/OP-24-00901 39761486 PMC12228835

[36] Chopra S , Kamdar D , Tulunay Ugur OE , et al. Factors predictive of severity of osteoradionecrosis of the mandible. Head Neck. 2011;33(11):1600‐1605. 10.1002/hed.21654 21484922

[37] Ben Dor B , Villa A , Hayes C , Alpert E , Shepard DS , Sonis ST . Financial burden of dental care among patients with head and neck cancer. JAMA Otolaryngol Head Neck Surg. 2024;150(9):811‐818. 10.1001/jamaoto.2024.2260 39088224 PMC11295059

[38] Graboyes EM , Ellis MA , Li H , et al. Racial and ethnic disparities in travel for head and neck cancer treatment and the impact of travel distance on survival. Cancer. 2018;124(15):3181‐3191. 10.1002/cncr.31571 29932220 PMC6097948

[39] Perlow HK , Ramey SJ , Cassidy V , et al. Disparities in adherence to head and neck cancer follow‐up guidelines. Laryngoscope. 2019;129(10):2303‐2308. 10.1002/lary.27676 30582620 PMC7757086

[40] Yarn C , Wakefield DV , Spencer S , Martin MY , Pisu M , Schwartz DL . Insurance status and head and neck radiotherapy interruption disparities in the Mid‐Southern United States. Head Neck. 2020;42(8):2013‐2020. 10.1002/hed.26128 32141652

[41] Beeler WH , Bellile EL , Casper KA , et al. Patient‐reported financial toxicity and adverse medical consequences in head and neck cancer. Oral Oncol. 2020;101:104521. 10.1016/j.oraloncology.2019.104521 31877502 PMC7008081

[42] Ma SJ , Iovoli AJ , Attwood K , et al. Association of significant financial burden with survival for head and neck cancer patients treated with radiation therapy. Oral Oncol. 2021;115:105196. 10.1016/j.oraloncology.2021.105196 33578203 PMC10353569

[43] Guerriero MK , Redman MW , Baker KK , et al. Racial disparity in oncologic and quality‐of‐life outcomes in patients with locally advanced head and neck squamous cell carcinomas enrolled in a randomized phase 2 trial. Cancer. 2018;124(13):2841‐2849. 10.1002/cncr.31407 29669181

[44] Lee A , Shah K , Chino F . Assessment of parking fees at National Cancer Institute‐designated cancer treatment centers. JAMA Oncol. 2020;6(8):1295‐1297. 10.1001/jamaoncol.2020.1475 32672809 PMC7366280

