# Supplementary material for: Implementation Mapping to Identify Strategies to Increase Timely Postoperative Radiotherapy Initiation for Head/Neck Cancer
Source: Otolaryngol Head Neck Surg. 2025 Apr 30;173(1):288–98. doi: 10.1002/ohn.1268 (PMC12207378; doi:10.1002/ohn.1268)
Supplement: Supplementary file 1 — Supplement: Anonymous 15‐item survey to assess barriers and facilitators to PORT initiation developed by study team members and fielded to members in the Departments of Radiation Oncology, Otolaryngology, and Medical Oncology. [file OHN-173-288-s001.pdf]

Optimizing Surgical/Radiation Oncology Workflow

We'd like to understand factors impacting timely initiation of post-operative radiation therapy (PORT) for head/neck cancer patients. Please answer the following brief questions. Your answers will be anonymous, and we will only review data in aggregate.

1. Mr. Smith is a 62 year-old male who recently underwent surgery for an oral cavity cancer. His pathology returned as an aggressive cancer (pT4aN2b, with perineural invasion, lymphovascular invasion, and multiple lymph nodes involved). This case was discussed in head/neck tumor board and recommendation was for post-operative radiation therapy.

   Please rate your agreement with the following statement: It is very important for patients like Mr. Smith to start radiation therapy within 6-weeks of completing surgery.

1	2	3	4	5

Strongly Disagree — Strongly Agree

2. In November 2021, the American College of Surgeons/CoC approved the first quality metric for head and neck oncology: time to initiation of postoperative radiation therapy less than or equal to 6 weeks for patients with surgically managed head and neck squamous cell carcinoma.

   How often do you believe patients such as Mr. Smith successfully start radiation within the 6-week recommended timeframe at NM?

- ○ 0-19%
- ○ 20-39%
- ○ 40-59%
- ○ 60-79%
- ○ 80-100%

3. When patients complete surgery, there are many steps that must be completed prior to starting post-operative radiation: completing surgery, recovering, seeing the surgeon post-operatively, referral, tumor board discussion, dental clearance, consult, simulation, time for planning, quality assurance, and treatment start.

   How seamless do you feel the overall process is?

1	2	3	4	5

   Very organized, little effort needed to start on time — Hectic, significant effort needed to start on time

4. How often do you feel your team is scrambling in hectic, last-minute, or unplanned ways to get a patient in for initiation of postoperative radiation therapy (PORT) in a timely fashion?

   - ○ Never/Almost never
   - ○ Rarely
   - ○ Sometimes
   - ○ Usually
   - ○ Almost Always/Always

5. What issues do you feel contribute to a delay in post-operative radiation start time? (Please select all that apply)

   - ☐ Timing of referral
   - ☐ Dental clearance
   - ☐ Post-operative complications
   - ☐ Receiving final pathology findings from surgery
   - ☐ Tumor board timing
   - ☐ Access to new radiation oncology consult appointment
   - ☐ Access to radiation therapy simulation (planning) scans
   - ☐ Sufficient time required to create radiation plan (for dosimetry)
   - ☐ Access to room/time on radiation machines to start treatment
   - ☐ Communication between teams
   - ☐ Other

6. What issues do you feel are within your circle of control and could be modified to improve timely initiation of post-operative radiation therapy? (Please select all that apply)

- ☐ Timing of referral
- ☐ Dental clearance
- ☐ Post-operative complications
- ☐ Receiving final pathology findings from surgery
- ☐ Tumor board timing
- ☐ Access to new radiation oncology consult appointment
- ☐ Access to radiation therapy simulation (planning) appointment
- ☐ Sufficient time required to create radiation plan (for dosimetry)
- ☐ Access to room/time on radiation machines to start treatment
- ☐ Communication between teams
- ☐ Other

7. Which aspects of workflow are currently working well and facilitate timely initiation of post-operative radiation therapy? (Please select all that apply)

- ☐ Timing of referral
- ☐ Dental clearance
- ☐ Post-operative complications
- ☐ Receiving final pathology findings from surgery
- ☐ Tumor board timing
- ☐ Access to new radiation oncology consult appointment
- ☐ Access to radiation therapy simulation (planning) scans
- ☐ Sufficient time required to create radiation plan (for dosimetry)
- ☐ Access to room/time on radiation machines to start treatment
- ☐ Communication between teams
- ☐ Other

8. When scrambling does occur to get patients started on timely post-operative radiation therapy, how does this show up? (Please select all that apply)

- ☐ Excessive messages between staff
- ☐ Excessive messages/calls to patient/family
- ☐ Overbooking for clinic or simulation times
- ☐ After hours work by team members
- ☐ Stressful or rushed workflows
- ☐ Other

9. How well do you feel the following teams work efficiently together?

	Poor	Margi nal	Satisfa ctory	Good	Optim al
Considering ONLY my own department, my team (MD/APP/RN/RTT/PTC) works efficiently and effectively together	○	○	○	○	○
Considering the ENT and RadOnc teams together, I believe our teams work efficiently and effectively together	○	○	○	○	○

10. How much do you agree or disagree with the following statements?

	Strongly disagree	Disagree	Neutral	Agree	Strongly Agree
I feel disappointed in myself or my team over failing to initiate radiation therapy within the recommended 6-week timeframe.	○	○	○	○	○
I feel like I have less control over my clinical care for patients when scheduling challenges occur (i.e. delayed PORT initiation)	○	○	○	○	○

11. Please indicate the extent to which you agree with the following statement:

	Never	Few Times a Year	Once a Month or Less	Few Times a Month	Once a Week	Few Times a Week	Every Day
I feel burned out from my work.	○	○	○	○	○	○	○

12. Are there are other aspects of our workflow related to achieving appropriate adjuvant radiation start time that could be improved or that are currently working well that you'd like to see continue?

13. What department do you work in?

- ○ ENT
- ○ Radiation Oncology
- ○ Other

14. What is your role?

- ◯ Nurse/APP
- ◯ Physician
- ◯ Radiation Dosimetrist or Therapist
- ◯ Resident/Fellow
- ◯ Other

15. Would you be interested in potentially participating in a focus group to brainstorm ideas to improve this process? If so, please write your name here and a member of our team will reach out to you.

This content is neither created nor endorsed by Microsoft. The data you submit will be sent to the form owner.

Microsoft Forms
